# First Evidence from Sri Lanka for Subphenotypic Diversity within *L. donovani*-Induced Classical Cutaneous Leishmaniasis

**DOI:** 10.1155/2021/3537968

**Published:** 2021-01-27

**Authors:** Yamuna Siriwardana, Bhagya Deepachandi, Sudath Weerasinghe, Nadira Karunaweera, Chandanie Udagedara, Wipula Warnasuriya, Ranthilaka R. Ranawaka, Indira Kahawita

**Affiliations:** ^1^Department of Parasitology, Faculty of Medicine, University of Colombo, Colombo 00800, Sri Lanka; ^2^Teaching Hospital, Kandy 20000, Sri Lanka; ^3^Teaching Hospital, Kurunegala 60000, Sri Lanka; ^4^Teaching Hospital, Kalutara 12000, Sri Lanka; ^5^Base Hospital, Homagama 10200, Sri Lanka

## Abstract

Sri Lanka reports a large focus of *Leishmania donovani*-induced cutaneous leishmaniasis (CL) with CL as the main clinical entity. Two independent, long existed, and clinicoepidemiologically different transmission foci in the northern region (NR) and southern region (SR) were recently reported. Current project is an extension to this previous study. Clinical diversity within a profile of classical cutaneous leishmaniasis (CCL) in a focus of *L. donovani*-induced CL is described for the first time. Patients with laboratory confirmed CCL (*n* = 550) from NF and SF were evaluated. Lesions in both foci were found to have all classical developmental stages (small and large nodules, ulcerating nodules, and ulcers) and other identified changes (multiplication, ulceration, and enlargement). Main difference was in the proportions of lesions progressing in to each different stages, proportions of lesion undergoing the main changes, and in timing of these changes during the course of a lesion. Northern focus reported a smaller proportion of lesions showing enlargement and ulceration, and a longer period of time was also required for these changes when compared to same in southern focus. In northern focus, most lesions remained small and nonulcerating and showed a higher tendency to multiply while most lesions reported in southern focus enlarged and ulcerated rapidly and remained single. Current study also evidenced a wider spectrum in the rate and pattern of progression of a skin lesion and high individual variation which could mask these region-based differences. Parasitic, vector-related, or a host etiology is suggested. Slow progressing nonulcerating infections in North may be the result of a well-adopted parasite strain that coevolved with its host for a long period while inducing only a minimal host response. This could be one among many reasons for previously observed silent expansion in northern focus while southern focus remained more confined and stable over time. Small nonprogressive, nondisturbing lesions can play a major role as silent parasite reservoirs in a community. In addition, the laboratory detection rate declined significantly when lesions multiplied and enlarged indicating the need for early laboratory confirmation. Usefulness of identified features in clinical screening and management needs to be considered.

## 1. Introduction

Leishmaniasis is a neglected tropical disease with increasing case burden in many countries and emerging new foci [1–7]. Clinically, apparent human infection presents in three main forms, cutaneous (CL), visceral (VL), or mucocutaneous (MCL), mainly depending on the causative species that are multiple within the genus *Leishmania*. Visceral infection is traditionally caused by *Leishmania donovani.* Untreated VL often leads to death. Indian subcontinent carries a huge burden of VL in the world. There is a regional drive for *L. donovani* control in which researchers have faced many difficulties [8].

*L. donovani*-induced CL is an increasingly reported new clinical entity in many countries. Sri Lanka reports a large focus of *L. donovani*-induced leishmaniasis with CL that remains as the main clinical entity over the past two decades [9–17]. Regional variation in the disease transmission profile in this focus was first indicated many years ago [13]. Subsequently, this focus reported many complexities associated with CL. Time-dependent microchanges were within the profile of classical CL (CCL) [16], and a separate entity of atypical cutaneous lesions [18], high CL-associated seroconversion rates [19, 20], and poor treatment response [21] together with emergence of MCL and VL [22–24] favored this observation. Northern and SR of the country were identified as main disease reporting areas [16, 25]. Subsequently, the two foci were identified to present independent, long exited, and clinicoepidemiologically different profiles [26]. Disease focus in the northern Sri Lanka reported young adult males (20-40 years old) with more nonulcerative lesions that remained small [26] over the time. This study provided primary evidence for possible two different phenotypes in Sri Lanka and evidenced for an underlying parasitic etiology.

In-depth information on clinical diversity enhances early case detection and facilitates decision on treatment options. However, information on the clinical profile of dermotropic *L. donovani* are still scarce in the world. Existing few reports also indicate considerable variation among patients or affected settings [27, 28]. Clinical markers were previously described only for a countrywide general profile of CL in Sri Lanka [14].

Due to the distinct nature of the causative organism in Sri Lanka and the long-term presence of independent transmission foci, possible differences in clinical patterns and their outcomes cannot be disregarded without proper evidence. Current project is an extension to the previously report region-dependent clinical variation of CL [26] and aimed at further examining the nature of clinical diversity.

## 2. Materials and Methods

Patients presented to the institution (2004-2013) with clinically suggestive locally acquired CCL were investigated by parasitological methods during routine screening (light microscopy of the lesion material or in vitro culture samples followed by the genus-specific PCR assay on microscopy negative cases) [29, 30]. A continuing database was developed in SPSS v20.0 with regard to clinical and investigation data during the study period. Patients (*n* = 550) presented from northern and southern disease foci with CCL were filter selected and further evaluated [26]. Randomly selected first lesion was counted in cases with multiple lesions. Missing and doubtful information was excluded case-wise or lesion-wise.

**Figure 1:**
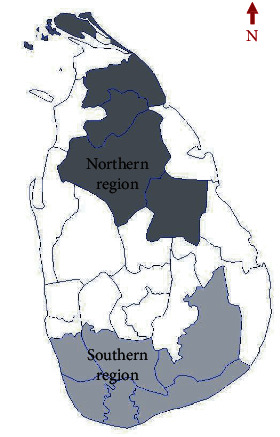
Northern and southern regions of Sri Lanka. The northern region included five districts from the North, and the southern region included six districts from South [26].

Working definitions were developed for data analysis purposes and interpretation of results, i.e., *primary lesion/typical onset*: skin nodule measuring <1 cm at maximum diameter and without lesion or skin discoloration [18]; *size*: maximum diameter of the observable lesion measured to the closest centimeter excluding visible induration; *early lesion*: a lesion of ≤3 months of duration according to patient's history; *chronic lesion*: a lesion of ≥12 months duration according to patient's history; *silent lesion*: a lesion that remains nonulcerative after 9 months of duration according to patient's history; *classical cutaneous leishmaniasis lesions*: small nodules (<1 cm diameter), larger nodules, ulcerating nodules, or completed ulcers [18]; *ulcerative lesions* (UT): lesions with disintegration of overlying epithelium (viz., ulcerating nodules, ulcerating plaques, and complete ulcers) [26]; *nonulcerative lesions* (NUT): lesions which were not accompanied by disintegration of overlying epithelium (viz., papules, nodules, and plaques) [26]; *northern region* (NR): five districts (i.e., Anuradhapura, Jaffna, Mullativu, Polonnaruwa, and Vavuniya) (Figure 1) [26]; and *southern region* (SR): six districts (Galle, Hambantota, Kalutara, Matara, Moneragala, and Ratnapura) (Figure 1) [26].

### 2.1. Analysis of Individual Variation

Region-dependent differences were analyzed by considering individual variations of rate of progression of skin lesions. Skewed data which showed high variations were identified and excluded from analysis using descriptive statistics. Differences in rate of progression of skin lesions between North and South foci were analyzed using two-way or three-way ANOVA. Rate of progression was assessed in terms of duration taken for ulceration, enlargement, and multiplication of a lesion. Violation of homogeneity of variance was assessed using Levene's test.

### 2.2. Ethical Aspects

Ethical clearance for the study was obtained from the Ethics Review Committee of Faculty of Medicine, University of Colombo, Sri Lanka.

## 3. Results

A total of 550 laboratory confirmed CCL lesions reported from North (*n* = 163) or South (*n* = 387) were considered for the clinical type analysis. A clear majority of them were first time presenters to the health sector (96.7%, *n* = 532/550). Northern focus reported 65% (106/163) nonulcerative lesions while southern focus reported only 43.6% (169/387) nonulcerative lesions.

### 3.1. Early and Chronic Lesions

Approximately, a half of early lesions ulcerated (49.1%, *n* = 112/228) and over 1/3^rd^ of them enlarged to a size of >2 cm (37.3%, *n* = 85/228). However, only 1/10^th^ of early lesions multiplied (11.0%, *n* = 25/228). In contrary, a higher proportion of chronic lesions remained nonulcerative (60.0%, *n* = 21/35) and nonenlarging (<2 cm), (51.4%, *n* = 18/35). Proportion of multiple lesions observed in this group was double that of early lesions (20.0%, *n* = 7/35 vs 11.0%, *n* = 25/228) (Table 1).

Early lesions were commonly observed in patients over 20 years (79.8%, *n* = 182/228 vs 20.2%, *n* = 46/228) while more chronic lesions were seen in younger individuals and young adult populations (37.1%, *n* = 13/35 and 45.7%, *n* = 16/35, respectively) (Table 1). Lesion-associated itchiness was more pronounced in early lesions (17.8%, *n* = 16/90 vs 4.0%, *n* = 1/25).

### 3.2. Progressive Lesions and Silent Lesions

Lesions ulcerated at early duration (progressive lesions) were compared with those remained nonulcerative for a longer period of time (≤9 months) (silent lesions).

Progressive lesions were nearly six times more likely to occur in the South as compared to the North (84.8%, *n* = 95/112 vs 15.2%, *n* = 17/112) (Table 2). In contrast, silent lesions were seen in approximately equal proportions in both regions (Table 2). The younger age group (<20years) was more likely to report silent lesions as compared to progressive lesions (15/44: 34.1% vs 22/112: 19.6%) (Table 2). There was no association between progressive or silent nature of lesions with gender or site of lesion. Both types of lesion occurred on the exposed body areas (data not shown).

Progressive lesions remained single in a clear majority (92.9%, *n* = 104/112) while silent lesions were three times more likely to multiply (22.7%, *n* = 10/44 vs 7.1%, *n* = 8/112) (Table 2). Nearly half of progressive lesions have also enlarged (44.6%, *n* = 50/112) while only 1/5^th^ (22.7%, *n* = 10/44) of silent lesions showed lesion enlargement. A change in rounded shape of a lesion was also observed in association with early ulceration (52.7%, *n* = 59/112 vs 25.0%, *n* = 11/44). Progressive lesions also showed color changes in almost all the cases while 15.9% (*n* = 7/44) of silent lesions did not show discoloration of lesion.

Erythema and hyperpigmentation were the commonly associated color changes of both types. Possibility for sporotrichoid spread was four times higher in lesions that remained silent while satellite spread was more common in early ulcerated lesions. Squamation and inflammatory reaction of the surrounding skin subsided with time (48.3% to 20.0% and 17.9% to 8.0%, respectively).

Local spread via satellite lesions was an early feature (14.3%) while sporotrichoid spread was more likely to occur in late lesions (Table 2). Squamation, inflammation, and hyperpigmentation changes were observed in early lesions more frequently when compared to late lesions (48.3%, 17.9%, 14.3% vs 20.0%, 8.0%, 4.0%).

### 3.3. Course of Skin Lesions in Northern and Southern Foci

**Figure 2:**
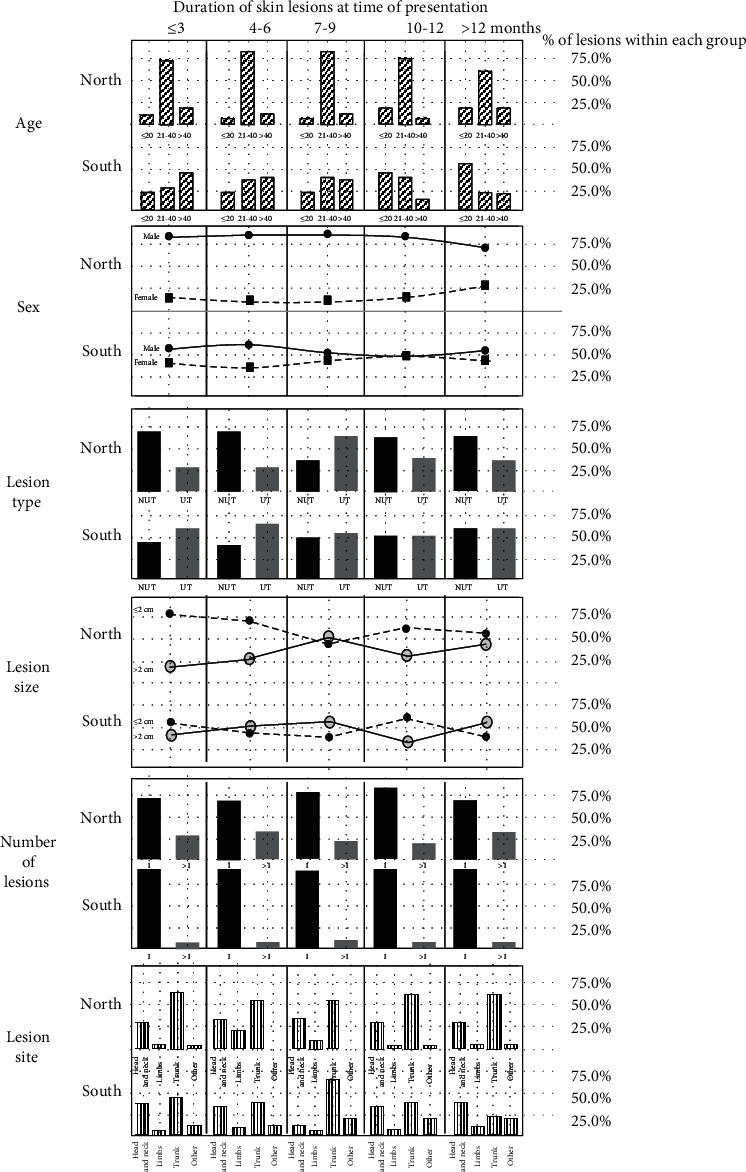
Course of skin lesions in the North and South. Percentages of lesions within each group are shown.

Proportion of young adults (21-40 years) reporting lesions at different durations remained the majority and varied from 63.2% to 82.4% in the North while in SR, patients at a wider age group reported lesions at all different durations (Figure 2). NR reported more males as compared to the South (Figure 2). Approximately 12.2%-26.3% females from the North and 38.3%-50.0% females from the South were reported at different durations. These age and sex patterns remained constant with early and late presentations of patients.

At any given duration including early and late durations of a skin lesion, majority of lesions remained nonulcerated in NR (35.3%-71.4%) while in the southern region, most lesions have started ulceration (43.8%-61.7%). Also, proportion of ulcerated lesions was markedly higher in the South than in the North at any given duration (Figure 2). Proportion of small lesions of different durations remained high in northern Sri Lanka (47.1%-78.9%) while it remained relatively low (43.2-66.7%) in the South. However, proportion of enlarged lesions was markedly higher in the South than the proportion reported from the North throughout the course of a lesion (Figure 2).Only a small proportion of lesions showed multiplication at a given time during the course of a lesion both in the North and South with a relatively high rate in the North (19.0%-32.7%) as compared to same in the South (5.8%-8.1%), (Figure 2). Both early and late skin lesions occurred mainly on distal limbs in both regions (Figure 2).

### 3.4. Enlargement, Multiplication, and Ulceration of a Skin Lesion

**Figure 3:**
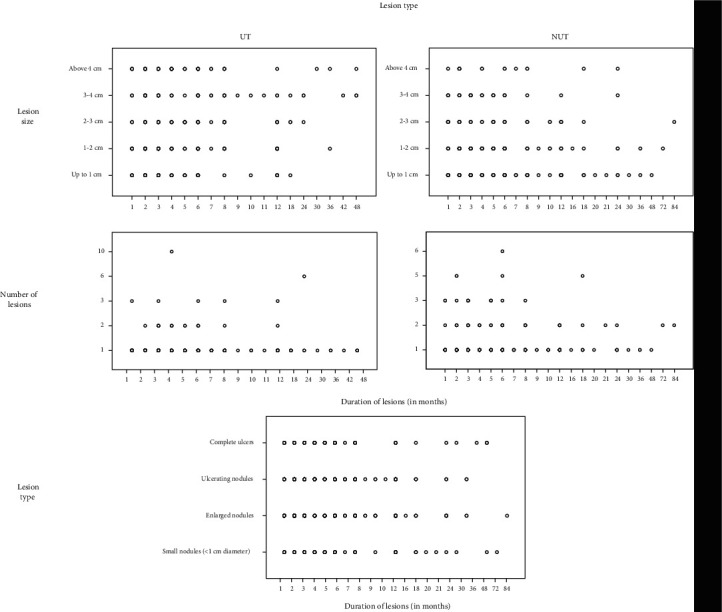
Individual variations of lesion features. Lesion multiplication, enlargement, and developmental progression over the time are shown.

During the development of a lesion, multiplication, enlargement, and ulceration were observed in different proportions and at different durations of a lesion (Figure 3). Nearly 14.1% (*n* = 11/78) of small (≤1 cm) lesions showed multiplication. However, in NR, a higher proportion of small lesions multiplied (21.9%, *n* = 7/32) while that of SR was only 8.7% (*n* = 4/46) (Table 3). This pattern remained almost unchanged with enlargement of a lesion. Among enlarged lesions, multiplication was seen only in NR while there were no large multiplied lesions in SR in this data set.

More than 1/3^rd^ (35.9%) lesions showed ulceration before they enlarge (≤1 cm) and thereafter, this was increasingly observed with lesion enlargement. However, among small lesions, only a small proportion ulcerated in the North (21.9%, *n* = 7/32) while a higher proportion ulcerated in SR (45.7%, *n* = 21/46). This tendency was constantly observed with lesion enlargement.

### 3.5. PPRs

The parasite positive rate (PPR) declined significantly when lesions multiplied to cause ≥3 lesions as compared to single or two lesions (78.6% vs 53.8%, *p* < 0.05) and in enlarged lesions (>2 cm) as compared to smaller (≤2 cm) lesions (79.7% vs 86.7%).

Individual variation:

### 3.6. Lesions over 18 Months

Most of these lesions remained nonulcerative (58.3%) (Figure 3). Some of them had an atypical onset (7.3%) as compared to those who had typical onset (1.7%), and trunk lesions were more likely to be long standing (12.5% vs 7.8% of <18 months lesions).

Though lesion ulceration, enlargement, and multiplication demonstrated an equal variance of duration among the North and South (*p* > 0.005 of Levene's test), rate of progression of skin lesions showed significant difference between the North and South (*p* < 0.05) (Figure S1).

Both NUT and UT lesions in the North showed a significantly higher mean duration compared to that in the South (Figure S1). Also, single lesions showed significantly high mean duration in the North compared to that in the South (Figure S1). Number of nonulcerative multiple lesions were high in the North [*n* = 25/92 (27.2%) from total NUT lesions] compared to that of the South [*n* = 14/160 (8.8%) from total NUT lesions], though they had low mean duration compared to multiple lesions in the South (Figure S1). Lesions in the North showed significantly high mean duration taken for lesion enlargement compared to lesions in the South (Figure S1).

## 4. Discussion

Clinical diversity within CCL caused by *L. donovani* is described for the first time.

**Figure 4:**
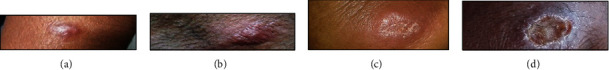
Skin lesions in patients with classical cutaneous leishmaniasis: (a) a small nodule (<1 cm diameter), (b) an enlarged nodule, (c) an ulcerating nodule, and (d) a complete ulcer.

CCL remained the main clinical form in Sri Lanka, though a proportion of lesions developed into atypical stages [18]. Current project demonstrated that both profile and course of a skin lesion in northern and southern foci are similar, and lesions in both sites belonged to one of the traditionally known stages of lesion development (Figure 4). Most lesions remained single and occurred mainly on exposed body areas which could probably be due to the clothing patterns in the community. Region-based difference was mainly related to the rate of progression of lesions and demographic features in the two foci.

A wider spectrum in the pattern of progression of CCL with an early ulcerative category and a chronic nonulcerative category of lesions at either end was also identified. Both types were more frequently seen in but not confined to a one geographical location. Time required for a lesion to progress or demonstrated changes also showed a wide spectrum.

Multiplication, enlargement, and ulceration are already known as lesion development-associated changes. Order in which these changes occurred was not constant in all individuals. However, a tendency for an ulcerated lesion to remain single and enlarge and a tendency for nonulcerative lesions to remain small and multiply were common observations. Evidence for lesion progression (multiplication, enlargement, and completed ulceration) and local spread (satellite spread and sporotrichoid spread) also seem to start early in UTs. Original rounded shape was retained in a half of early ulcerated lesions. Altered lesion color (erythema, pigmentation changes), altered skin color, and skin squamation are also associated with UTs. Secondary infections can affect the severity and size of the cutaneous lesions, though this was not a clinically observed issue in the study group, except for crust formation in some lesions. Crust was carefully removed at the time of investigations.

Descriptions on the course of leishmanial skin lesions are scarce in literature. Phenotypic data on *L. donovani*-induced CL is even more scarce. Papular lesion onset of *L. donovani*-induced CL was also reported in Turkey [31]. Also, clinical manifestations of *L. donovani* infections showed noduloulcerative plaques, hypopigmented erythematous nodules, or papules from endemic foci in India [28, 32].

In general, study findings favored a slow silent nature in progression in the North while lesions in southern focus seem to progress rapidly. This observation was applicable to both ulcerative and nonulcerative lesions in southern and northern foci. In addition, high individual (case dependent) variation in the rate of progression of a skin lesion was also observed. However, some patients would have acquired the infection while travelling or working in an area in another focus, creating a masking effect on the region-dependent differences.

Slow progressing lesions in the North indicate a more stable host-parasite relationship leading to silent expansion of the disease focus. This could be an underlying reason for the previously observed silent expansion of the northern focus while southern focus remained more confined and stable over the time [26, 33]. Slow progressing nonulcerating infections in the North may be the result of a well-adopted parasite strain that coevolved with its host for a long period of time while inducing only a minimal host response. In contrary, the infections that occur in southern focus seem to exert a more robust host response. Vectorability of the sandfly populations, differences in human or parasite genetics, and immunological and endocrinological aspects need to be studied in order to understand the underlying etiology for region-based differences. Size, lesion type, and number of lesions have shown to be species-dependent [34]. However, some other studies have shown that diversity of clinical presentations of CL could not be explained by species differences [35]. Complex association between the genetics of the parasite and the host immune response had also been suggested [36]. Relevance of host genetics, ethnicity, local exacerbated inflammatory, and host immune responses in development of skin lesions have been reported [37–39]. Host genes contributing towards susceptibility, resistance, or protection against different pathologies have been recorded in American cutaneous leishmaniasis [39, 40]. Also, sandfly saliva may enhance the development of skin lesions by inhibiting some immune functions of the host macrophages and by shortening the generation time of dividing intracellular amastigotes [41].

Wider age distribution and less marked gender preponderance seen in the South and concentration of cases in the 21-40 year group and male preponderance in the North had been observed since long [26]. Male preponderance in *Leishmania* infection could mostly be due to behavioral factors including occupational exposure and other social behavior patterns. Agricultural work, military activities in rain forests, and outdoor activities during times of maximum vector activity may affect male preponderance of disease [19, 42]. Presence of a more outdoor behaving vector population in the North could be one reason for current observation. Vector abundance in peridomestic environments in SR may have exposed more females and younger and elderly groups to infection as reported previously as well [13, 43]. However, high level of testosterone in young adults also increases susceptibility to *Leishmania* infections in young adults [44, 45].

Study findings also indicated important clinical implications. Small, nonpainful, and nontender lesions can often go unnoticed or can be ignored by individuals resulting in poor or late self-referrals that facilitate silent parasite reservoirs in a community. The parasite detection rate was low in multiplied and enlarged lesions, probably due to well-established host immune mechanisms leading to a reduction of parasite numbers. This finding indicate that the need for early investigation is leishmaniasis. Observations on lesion progression will be useful during intra or perilesional drug administration. This route may be more effective if started early before multiplication. Evidence for local spread in ulcerated lesions showed a variable tendency. Possibility for fast and slow progressions in southern and northern regions, respectively, could be expected. Treatment of ulcerating lesions may minimize development of undesired scars while early treatment of small nonulcerative lesions could minimize the parasite reservoir in the community. A relook at the previously developed clinical scoring system [14] may be now worthwhile based on regional differences. Though the possibility of visceralization or mucosal tissue localization was not indicated in this focus [46–48], this risk cannot be completely disregarded at least in a minority. Adequate patient follow-up is desirable.

## 5. Conclusions

This study provided further insight into previously observed region-based differences in the epidemiological profiles of CL in northern and southern Sri Lanka. Infections in both sites have similar profiles and follow a similar course of lesion development with different rates in progression. Slow progressing profile in northern focus indicate long-term coevolved and highly adopted parasite strains in this region and differences in host genetics or vectorability of sandflies. In addition, treatment response and sequalae of treated and untreated infections may be different in the two regions.

## Figures and Tables

**Table 1:** Comparison of clinical profile of early and late lesions.

Clinical characteristics		Early lesions (≤3 months)		Chronic lesions (>12 months)	
		Count	(%)	Count	(%)
Lesion type	Papular	31	(13.6)	9	(25.7)
	Nodular	85	(37.3)	12	(34.3)
	Ulcerating nodules	62	(27.2)	7	(20.0)
	Complete ulcers	50	(21.9)	7	(20.0)
	Total	228	(100.0)	35	(100.0)

Lesion size	≤2 cm	143	(62.7)	18	(51.4)
	>2 cm	85	(37.3)	17	(48.6)
	Total	228	(100.0)	35	(100.0)

Number of lesions	Single	203	(89.0)	28	(80.0)
	Multiple	25	(11.0)	7	(20.0)
	Total	228	(100.0)	35	(100.0)

Age in years	Up to 20	46	(20.2)	13	(37.1)
	21-40	91	(39.9)	16	(45.7)
	Over 40	91	(39.9)	6	(17.1)
	Total	228	(100.0)	35	(100.0)

Lesion itchiness^∗^	Reported	16	(17.8)	1	(4.0)
	Not reported	74	(82.2)	24	(96.0)
	Total	90	(100.0)	25	(100.0)

^∗^Missing data were excluded.

**Table 2:** Comparison of progressive (early ulcerating lesions: UTs) and silent lesions (late nonulcerating lesions: NUTs).

Clinical characteristics		Progressive lesions UTs of ≤3 months^∗^		Silent lesions NUTs of >9 months		*p* value^#^
		Count	(%)	Count	(%)	
Geographical origin	Northern region	17	(15.2)	21	(47.7)	≤0.001
	Southern region	95	(84.8)	23	(52.3)	
	Total	112	(100.0)	44	(100.0)	
Age (years)	Up to 20	22	(19.6)	15	(34.1)	0.002
	21-40	44	(39.3)	24	(54.5)	
	Over 40	46	(41.1)	5	(11.4)	
	Total	112	(100.0)	44	(100.0)	
Number of lesions	Single	104	(92.9)	34	(77.3)	0.006
	Multiple	8	(7.1)	10	(22.7)	
	Total	112	(100.0)	44	(100.0)	
Size of lesion	≤2 cm	62	(55.4)	34	(77.3)	0.011
	>2 cm	50	(44.6)	10	(22.7)	
	Total	112	(100.0)	44	(100.0)	
Shape of lesion	Rounded	53	(47.3)	33	(75.0)	0.002
	Shape altered	59	(52.7)	11	(25.0)	
	Total	112	(100.0)	44	(100.0)	
Color of lesion	No change	1	(0.9)	7	(15.9)	0.002
	Erythematous	74	(67.0)	25	(56.8)	
	Hyperpigmented	27	(24.1)	8	(18.2)	
	Hypopigmented	10	(8.9)	4	(9.1)	
	Total	112	(100.0)	44	(100.0)	
Other associations^∗∗^						—
Squamation of skin	Observed	14	(48.3)	5	(20.0)	
Sporotrichoid lesions	Observed	1	(3.6)	3	(12.0)	
Satellite lesions	Observed	4	(14.3)	2	(8.0)	
Skin hyperpigmentation	Observed	4	(14.3)	1	(4.0)	
Skin hypopigmentation	Observed	8	(28.6)	8	(32.0)	
Skin inflammation	Observed	5	(17.9)	2	(8.0)	

^∗^From the early lesion group, ^∗∗^data pertaining only to relevant categories within each variable are shown (*p* values were not calculated). ^#^*p* values were calculated using online statistical program, VassarStats (available on http://vassarstats.net/).

**Table 3:** Enlargement, multiplication, and ulceration of early lesions.

Geographical region	Clinical characteristics of lesions		Lesion size									
			≤1 cm		1-2 cm		2-3 cm		3-4 cm		>4 cm	
			Count	(%)	Count	(%)	Count	(%)	Count	(%)	Count	(%)
North	Lesion number	≤1	25	(78.1)	9	(69.2)	7	(100.0)	1	(33.3)	0	(0.0)
		>1	7	(21.9)	4	(30.8)	0	(0.0)	2	(66.7)	2	(100.0)
		Total	32	(100.0)	13	(100.0)	7	(100.0)	3	(100.0)	2	(100.0)
	Lesion type	NUT	25	(78.1)	9	(69.2)	3	(42.9)	2	(66.7)	1	(50.0)
		UT	7	(21.9)	4	(30.8)	4	(57.1)	1	(33.3)	1	(50.0)
		Total	32	(100.0)	13	(100.0)	7	(100.0)	3	(100.0)	2	(100.0)

South	Lesion number	≤1	42	(91.3)	48	(92.3)	22	(91.7)	27	(100.0)	22	(100.0)
		>1	4	(8.7)	4	(7.7)	2	(8.3)	0	(0.0)	0	(0.0)
		Total	46	(100.0)	52	(100.0)	24	(100.0)	27	(100.0)	22	(100.0)
	Lesion type	NUT	25	(54.3)	22	(42.3)	12	(50.0)	11	(40.7)	6	(27.3)
		UT	21	(45.7)	30	(57.7)	12	(50.0)	16	(59.3)	16	(72.7)
		Total	46	(100.0)	52	(100.0)	24	(100.0)	27	(100.0)	22	(100.0)

Total	Lesion number	≤1	67	(85.9)	57	(87.7)	29	(93.5)	28	(93.3)	22	(91.7)
		>1	11	(14.1)	8	(12.3)	2	(6.5)	2	(6.7)	2	(8.3)
		Total	78	(100.0)	65	(100.0)	31	(100.0)	30	(100.0)	24	(100.0)
	Lesion type	NUT	50	(64.1)	31	(47.7)	15	(48.4)	13	(43.3)	7	(29.2)
		UT	28	(35.9)	34	(52.3)	16	(51.6)	17	(56.7)	17	(70.8)

## Data Availability

Data supporting conclusions of this article are included within the article. Other data has not been made available as it was not part of the ethics application and due to patient confidentiality.

## Supplementary Materials

Supplementary Materials Figure S1 Difference of rate of progression of skin lesions between the North and South. (a) Mean duration taken for lesion ulceration (UT: ulcerative type; NUT: nonulcerative type). (b) Mean durations taken for lesion ulceration and multiplication (>1 lesion) of nonulcerative lesions. (c) Mean durations taken for lesion ulceration and multiplication of ulcerative lesions. (d) Mean duration taken for lesion enlargement.
