# Supplementary figures and images for: First Evidence from Sri Lanka for Subphenotypic Diversity within L. donovani-Induced Classical Cutaneous Leishmaniasis
Source: Biomed Res Int. 2021 Jan 27;2021:3537968. doi: 10.1155/2021/3537968 (PMC7861938; doi:10.1155/2021/3537968)

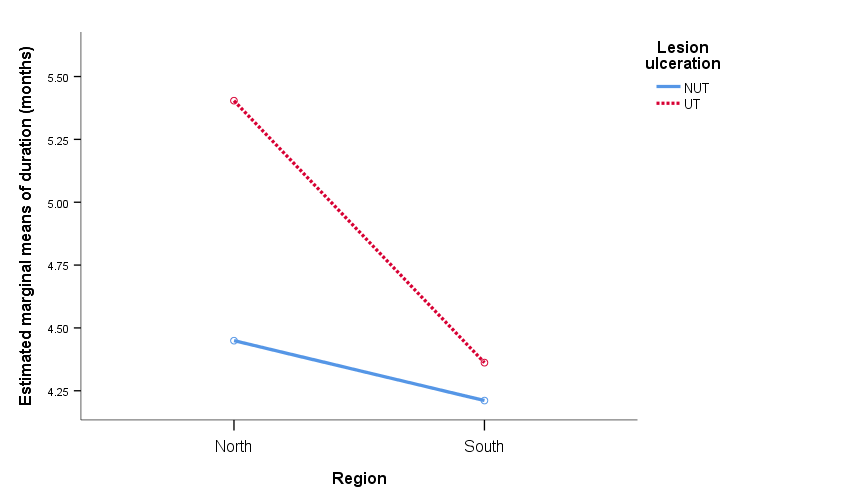


a


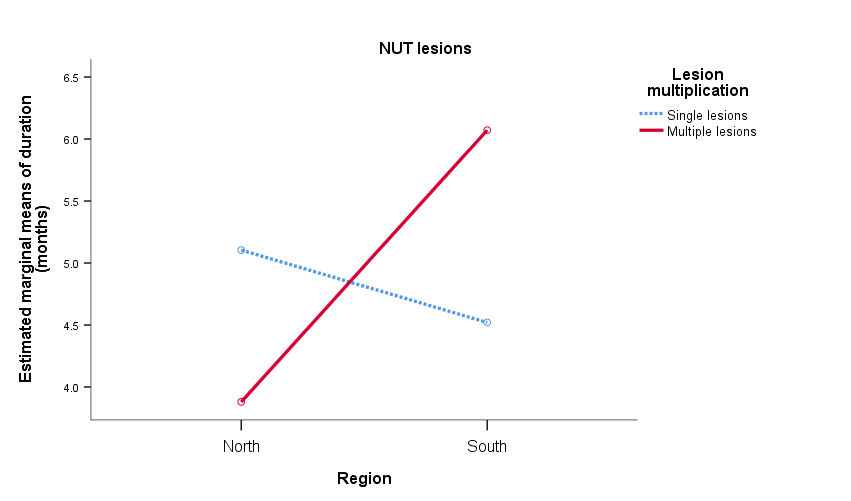

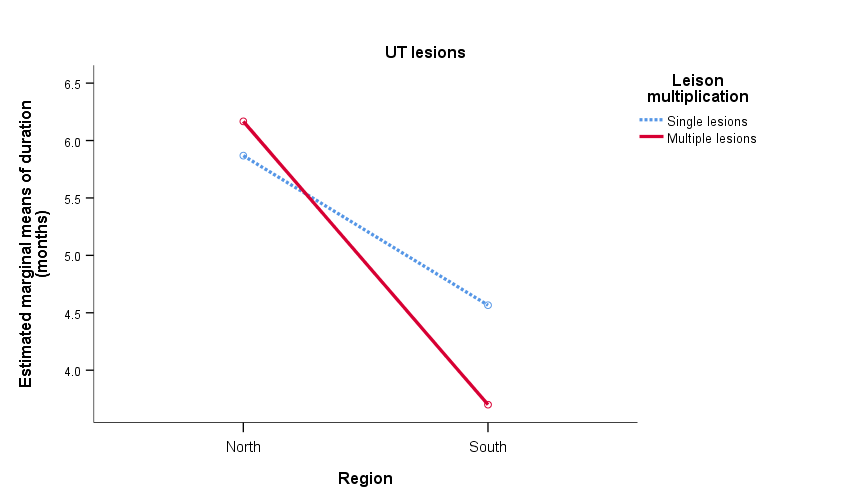

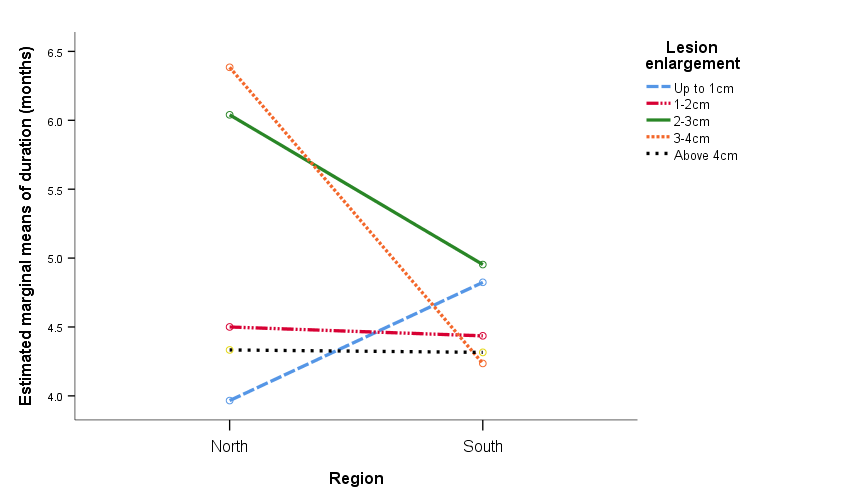


b

c

d

Supplement: Supplementary Materials — Figure S1 Difference of rate of progression of skin lesions between the North and South. (a) Mean duration taken for lesion ulceration (UT: ulcerative type; NUT: nonulcerative type). (b) Mean durations taken for lesion ulceration and multiplication (>1 lesion) of nonulcerative lesions. (c) Mean durations taken for lesion ulceration and multiplication of ulcerative lesions. (d) Mean duration taken for lesion enlargement. [file 3537968.f1.docx]
